# Efficient cutting stock optimization strategies for the steel industry

**DOI:** 10.1371/journal.pone.0319644

**Published:** 2025-03-28

**Authors:** Chattriya Jariyavajee, Suthida Fairee, Charoenchai Khompatraporn, Jumpol Polvichai, Booncharoen Sirinaovakul

**Affiliations:** 1 Department of Computer Engineering, Faculty of Engineering, King Mongkut’s University of Technology Thonburi, Bangkok, Thailand; 2 Independent researcher, Bangkok, Thailand; 3 Department of Production Engineering, Faculty of Engineering, King Mongkut’s University of Technology Thonburi, Bangkok, Thailand; 4 Graduate School of Management and Innovation, King Mongkut’s University of Technology Thonburi, Bangkok, Thailand; University of Birmingham, UNITED KINGDOM OF GREAT BRITAIN AND NORTHERN IRELAND

## Abstract

This study addresses a cutting stock problem in steel cutting industry by developing a mathematical model in which machine specifications and cutting conditions are constraints. The solution process involves three key steps: (i) Problem representation, where feasible cutting solutions are modeled based on pre-cut steel bars and customer orders, (ii) Problem space reduction, which reduces the problem space by eliminating suboptimal solutions and following manufacturer loss limits, and (iii) Optimal solution search, whereas the optimal solution is identified using a new Adaptive Pathfinding Optimization Algorithm. This algorithm combines a newly proposed Wandering Ant Colony Optimization with a brute force method, and uses specific conditions to determine which of these two approaches to be used to obtain the solution. The proposed algorithm can also be applied to other cutting stock problems, such as paper roll cutting, metal rod cutting, and wood plank cutting. The algorithm was applied to real customer orders in a steel manufacturer and showed significant benefits by reducing the number of planners from four to merely one person and decreasing the cutting planning time from six hours to under one hour. Additionally, the algorithm yields an average cost saving of USD 3.95 per ton, or 52.18% of the baseline.

## Introduction

The cutting stock problem (CSP) is a notable optimization challenge encountered in various industries, such as paper production, steel manufacturing, marble processing and construction. It involves the efficient utilization of large stocks to produce smaller components while minimizing waste (trim loss), meeting demands and adhering to specific cutting and production constraints [1–4].

To handle objectives with different parameters, such as cost, man-hours, and other production measures, researchers often normalized and combined them into one. Costs are common measures. Subsequently, all of these costs were combined as a single objective for optimization. For instance, Ma et al. [5] optimized the combined costs of setup and material waste. Pierini and Poldi [6] considered the setup, production and inventory holding costs of the intermediate, and final products and the material waste costs as multiple objectives. Oliveira et al. [7] integrated CSP with lot sizing and set various production costs, including cutting, setup and inventory holding costs, as objectives to be minimized. Recent studies on CSP often include production constraints, such as lot size, production period and cutting stage [4,8,9] as well as productivity issues, such as the production sequence to minimize the makespan and number of cutting patterns to reduce the setup time [10,11]. The theoretical difficulty of this problem is classified as NP-complete [12], and additional complexity arises from the integration of the CSP with the production requirements. Consequently, multi-objective CSPs have been researched.

In multi-objective optimization problems, the optimal solutions lie on a Pareto-optimal frontier, representing trade-offs among conflicting objectives. Hence, there exists no single solution that can simultaneously optimize all objectives; however, the solutions on the Pareto frontier compromise conflicting objectives. The decision-maker may select any Pareto optimal solution that suits the practical context of the problem. For instance, Aliano Filho, Moretti and Vaz Pato [10] aimed to reduce the trim loss and number of cutting patterns. These two objectives are conflicting because reducing trim loss usually leads to frequent variations in cutting patterns, thereby increasing the machine setup time. However, cutting patterns that require fewer machine setups often incur high trim loss. Ren et al. [13] minimized the trim loss rate and number of cutting patterns while cutting various sizes of reinforced concrete bars and maintaining structural requirements.

Standardizing multi-objective functions in the same unit, which involves multiplying individual objectives by scalar weights before adding them, has also been proposed. This approach, commonly known as the weighted-sum method [10,14], allows for objective prioritization. Compared to lower weights, higher weights assigned to objectives indicate greater importance; however, the weight values can be adjusted based on specific circumstances. For instance, Kim et al. [8] utilized the weighted-sum method to address window-frame production. Because the weighted sum method inherently considers trade-offs among objectives based on decision-maker-specified weights, solutions are evaluated using the weighted function of individual objectives instead of the Pareto frontier [15].

Optimally solving the CSP remains a challenge, particularly for large-scale instances. While exact methods exist, heuristics and approximate approaches are often recommended [12,16]. Gilmore and Gomory [17] pioneered the development of CSP solution approaches by reformulating the CSP as a relaxed linear program and used a simplex-based method to solve the relaxed problem; this method is known as column generation. Other heuristics, such as in [18], Armbruster presented a sequencing algorithm to generate cutting patterns that enable the algorithm to solve a continuous relaxation of CSP and derive the corresponding integer solutions. However, Rietz and Dempe [19] remarked that there could be large ‘gaps’ between the original and relaxed problems; therefore, the original problem should be solved directly.

Heuristic approaches solve the original CSP but obtain only near-optimal solutions. For instance, Aliano Filho, Moretti and Vaz Pato [10] proposed a heuristic that optimized several subproblems for a bi-objective CSP. Cerqueira, Aguiar and Marques [20] modified greedy heuristic to solve the test instances of CSP. Birgin, Romão and Ronconi [21] developed an advanced approach to solve the multi-period problem, in which leftovers from previous periods should be minimized. Araujo et al. [22] aimed to reduce the number of patterns needed for 1D precast beam production in multi-period planning, and developed size-reduction heuristics and focused more on promising subsets of variables in the exact model. There are commercial applications available to solve linear/non-linear optimization problems. However, for realistic situations, solvers such as CPLEX has limitation on insufficient memory and time consuming [23].

Metaheuristics have also been implemented in several studies. Taetragool, Sirinaovakul and Achalakul [24] introduced an adapted artificial bee colony algorithm to address the combinatorial optimization problem. Guo [25] modified the ant colony optimization (ACO) [26] with mutations to enhance the local search and solve the CPS in a bin-packing problem. Mellouli et al. [27] combined genetic algorithm with a mixed-integer linear programming model to solve a bi-objective CSP. Ravelo, Meneses and Santos [28] implemented a constructive heuristic, GRASP-based algorithm and an evolutionary algorithm for cutting stock test problems. Luo et al. [29] developed a randomized multi-start algorithm comprising a biased genetic algorithm, variable neighborhood search and grey wolf optimization to minimize the number of strips in the two-dimensional CSP of metal sheets. Ren et al. [13] applied a particle swarm optimization algorithm to solve the CSP of reinforced concrete bars. Pitombeira-Neto and Murta [30] reformulated the CSP as a stochastic optimization problem to account for dynamic inventory, and used a reinforcement learning based technique to approximate the solution.

In our case study, the problem is complicate in that there could be numerous stock bar lengths to be selected, many machine constraints to be satisfied, saw positions and engagement statuses to be determined, as well as many production measures to be optimized. We propose an effective approach to address the CSP in a steel manufacturer, while considering the complexity involved in varying cutting conditions and requirements. The CSP is formulated as a mathematical model that integrates machine specifications and customer orders as constraints. The problem space is systematically reduced by eliminating suboptimal solutions and applying manufacturer loss limit conditions. A proposed Adaptive Pathfinding Optimization Algorithm (APOA), combining the newly proposed Wandering Ant Colony Optimization (WACO) with the brute force method, is then employed to determine the optimal solution. The specific conditions dictate which algorithm is activated to find the best solution.

The remainder of this paper is organized as follows: The Problem Formulation section explains the CSP formulation as a nonlinear mathematical model that reflects the manufacturer’s objectives, customer requirements, and cutting machine specifications. The Solution Approach section describes the Problem representation, details the Problem space reduction, and then outlines the Search for the optimal solution using the proposed algorithm. The Result and Discussion section compares outcomes and evaluates the effectiveness of the algorithm in a real-world scenario. Finally, the Conclusion section summarizes the key findings and practical implications of the approach, with a discussion on its potential application to other cutting stock problems, such as paper roll, metal rod, and wood plank cutting.

## Problem formulation

The steel cutting process starts from steel blooms, which are rolled into bars to a pre-specified length and cross-section under elevated temperatures; these hot-rolled bars are referred to as precut bars. The two jagged ends of the precut bars are removed using a cutting machine, and the intermediate flat-sawn end bars have the intended rolling lengths. Thus, these bars are called the as-rolled length (ARL) bars, as described by the manufacturer. As per customer orders, these ARLs are cut into smaller-sized bars of specific lengths, which are the final products to be cooled in the cooling beds. Each cooling bed can accommodate a single size of the final product. In actual production, two of the eight cooling beds operate simultaneously. Hence, the number of operating cooling beds limits that of the final product sizes, which can be reduced from a given ARL to two sizes of any combination.

The steel grade, classified by the chemical composition of blooms, determines the grade of the ARL bars and final products. Different steel grades, cross-sectional areas and dimensions determine different steel series. Currently, over 1,600 series are managed by the manufacturer. These series are available in the company’s catalogue, from which customers place orders by specifying the required final product sizes and total numbers. Each month, approximately one-fourth to one-third of these codes are ordered by customers and require cutting plans.

### Problem description

The CSP in this study required ARL bars of up to 24 different lengths per product group. A product group is a group of final products (from the same series) that are aggregated from several orders. This group comprises the lengths and quantities of the final products that must be cut. It normally contains up to six different lengths, with up to 6,000 final products.

The cutting process is assessed to minimize the following three objectives: trim loss, over rolls and cutting time. Trim loss is the unusable leftover material from the cutting process. Contrastingly, over rolls are leftover materials that are saleable because they are usually long enough to be cut into final products. These over rolls are stored in warehouses, waiting for unpredictable future orders, and are thus not desirable.

Cutting time is associated with productivity. Any pattern that requires a saw position change requires a longer time. These objectives are emphasized based on the prevailing production planning conditions. During periods of high customer order volumes, productivity takes precedence in ensuring timely delivery. Conversely, when there are fewer orders, minimizing trim losses to effectively reduce waste costs becomes critical. Reducing the over rolls to save inventory holding costs can be considered at any stage; however, its importance may vary based on space availability in the warehouse. The dynamic prioritization of these objectives reflects the adaptive nature of real-life cutting stock optimization. Here, the CSP is used to determine the cutting patterns that would ensue the minimization of these objectives.

### Cutting machine specifications

Several constraints are imposed by production machinery. In addition to the two operational cooling beds at the end of the cutting line, a cutting machine with three saws was installed to cut three pieces of the final product in a single feed. However, these pieces are subject to certain constraints, such as length limitations of the final products and removal of jagged ends, which can only be achieved using specific saws.

**Fig 1:**
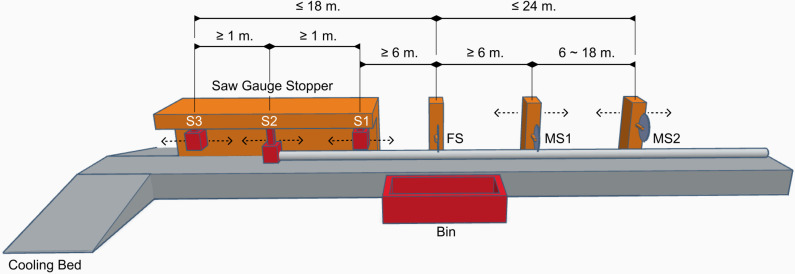
Schematic of the cutting machine.

Fig 1 shows the main cutting components of the cutting machine. It is assumed that the pre-cut (or hot-rolled) bars with jagged ends are transferred from right to left. The specific details of the cutting machine are as follows:

- The saw on the left, called the fixed saw (FS), is placed in a stationary position.
- Two movable saws are positioned to the right of FS. The saw on the immediate right of the FS, or the middle saw in the diagram, is referred to as the movable saw 1 (MS1). The other movable saw, i.e., the right saw in the diagram, is called the movable saw 2 (MS2).
- Three saw–gauge stoppers are placed to the left of FS with movable stoppers. The rightmost stopper (S1) is positioned 6–16 m away from the FS. The other stoppers (S2 and S3) must be at least 1 m away from the contiguous stoppers, whereas the leftmost allowable position is 18 m away from the FS. It is not necessary for all stoppers to be used in stopping the incoming bar in a feed; in fact, only one stopper is needed to engage in a feed.
- The area around FS is used to collect the trims into the trash bin. In the first feed of each cutting pattern, a jagged end is cut from the FS and situated to the left of the saw. In the last feed, the other jagged end is also cut by the FS and situated to the right side of the saw.
- When all the saws are used to cut a feed, the final product (excluding the jagged end) that is cut by the FS and is to the left of the saw is referred to as Cut 1 in the feed. The final product cut by FS and MS1 is referred to as Cut 2. Similarly, the final product cut by MS1 and MS2 is called Cut 3. From the diagram, Cuts 1, 2 and 3 must be 6–18 meters long where the length must not exceed 42 meters in total. Additionally, the combined lengths of Cuts 2 and 3 must not exceed 24 meters. However, the machine produces only one or two cuts when a few saws are disengaged during cutting. The cuts are labelled as Cuts 1 and 2 in a left-to-right manner. The product lengths of Cuts 1 and 2 are in the ranges of 6–42 and 6–24 m, respectively.
- The machine configuration consists of several stoppers and saws. Prior to production, all stopper and cutting saw positions are manually configured for each cutting pattern. If their positions remain unchanged and the machine merely engages or disengages the stoppers and saws, the maximum time required for a feed to transfer the ARL bar and be cut simultaneously by all saws is 55 s. Conversely, if any stopper or saw positions are altered in any feed, the average cutting time for that feed is approximately 600 s because these positions are manually adjusted.

The cutting pattern is constructed using multiple feeds and a specified ARL. Each feed consists of up to three cuts (1, 2 and 3). The ARL value is selected based on the manufacturer-provided ARL lengths. Numerous cutting patterns should be utilized to obtain the ordered products, and the final products are transferred and accumulated on the cooling beds.

### Mathematical model

#### Sets and indices

P Set of cutting patterns, where P = {1, 2, …, *P*}
L Set of product lengths, where L = {1, 2, …, *L*}
SSSet of stoppers and saws, where SS =  {S3, S2, S1, FS, MS1, MS2}
${ℱ}_{p}$Set of feeds in pattern *p*, where ${\text{ℱ}}_{p}$ = {1, 2, …, *F*_*p*_}
${\text{ℰS}}_{p,f_{p}}$Set of positions of engaging stoppers and saws in pattern *p* and feed $f_{p}$
*p* Index of cutting patterns in the cutting pattern set, where *p* ∈℘
*l* Index of product lengths in the product length set, where *l* ∈ℒ
*c* Index of the cuts in each feed, where *c*  ∈  {1,2,3}.
*s* Index of stoppers and saws, where *s* ∈SS
$f_{p}$Index of feeds in pattern *p*, where $f_{p}\in {\text{ℱ}}_{p}$

#### Parameters

*Z* Objective function
*CT* Cutting cost per second
$ARL_{p}$Length of an ARL to be cut with pattern *p*
$BW_{p}$Weight (in tons) of a steel bloom used in pattern *p*
$CS_{l}$Inventory holding cost of product *l*
$CW_{p}$Cost per unit weight based on pattern *p*
$PL_{l}$Length (in meters) of product *l*
$Q_{l}$Total demand or quantity of product *l* ordered by the customers
$WM_{p}$Weight (in tons) per length (in meters) of ARL to be cut based on pattern *p*
$w_{k}$Important weight of the objective *k*, where $k\in \left\{1,2,3\right\}$, $w_{k}\in \left[0,1\right]$ and $\sum_{k=1}^{3}w_{k}=1$

#### Decision variables

$q_{p}$Quantity of pattern *p* to be produced
$e_{p,f_{p}}^{s}$Engagement of stopper and saw *s* in pattern *p* and feed *f*_*p*_, valued as 1 when *s* is engaged and 0 otherwise.
$x_{p,f_{p}}^{s}$Position of stopper and saw *s* in pattern *p* and feed *f*_*p*_

#### Related variables

$OR_{l}$Over rolls of product *l*
$TM_{p}$Overall cutting time of pattern *p*
$TW_{p}$Trim loss weight (in tons) of pattern *p*
$ES_{p,f_{p}}^{i}$Position of engaging stopper and saws in pattern *p* and feed *f*_*p*_, where $i\in \left\{1,2,3,4\right\}$ and $ES_{p,f_{p}}^{i}\in {\text{ℰS}}_{p,f_{p}}$
$q_{l}$Quantity of product *l* made by any pattern, feed, or cut
$l_{p,f_{p}}^{c}$Cutting length (in meters) in pattern *p*, feed *f*_*p*_ and cut *c*
$t_{p,f_{p}}^{c}$Cutting time (in seconds) of pattern *p*, feed *f*_*p*_ and cut *c*
$m_{p,f_{p},c}^{l}$Indicating variable with value 1 when product *l* is cut by pattern *p*, feed *f*_*p*_ and cut *c*, and 0 otherwise

#### Cutting outcomes

The trim loss weight of the cutting pattern *p* is


$$TW_{p}=BW_{p}-WM_{p}\left(\sum_{f_{p}\in {ℱ}_{p}}\sum_{c=1}^{3}l_{p,f_{p}}^{c}\right)$$ (1)

The over rolls of a product length *l* is


$$OR_{l}=q_{l}-Q_{l}$$ (2)

The total time utilized by pattern *p* is


$$TM_{p}=\sum_{f_{p}\in {ℱ}_{p}}\text{max}\left\{t_{p,f_{p}}^{1},t_{p,f_{p}}^{2},t_{p,f_{p}}^{3}\right\}$$ (3)

#### Objective function


$$Minimize\;Z=w_{1}\sum_{p\in ℘}CW_{p}q_{p}TW_{p}+w_{2}\sum_{l\in ℒ}CS_{l}OR_{l}+w_{3}CT\sum_{p\in ℘}q_{p}TM_{p}$$ (4)

#### Constraints

The FS is the reference position for the stoppers and saws, with the assigned position of 0. The positions to the left and right of the FS correspond to negative and positive numbers, respectively. As shown in Fig 1, the positions of the stoppers and saws are denoted as follows:


$$x_{p,f_{p}}^{S1}\in \left\{-16\text{to}-6\right\},x_{p,f_{p}}^{S2}\in \left\{-17\text{to}-7\right\},x_{p,f_{p}}^{S3}\in \left\{-18\text{to}-8\right\},\forall p,\forall f_{p}$$ (5)

$$x_{p,f_{p}}^{FS}=0,x_{p,f_{p}}^{MS1}\in \left\{6\text{to}18\right\},x_{p,f_{p}}^{MS2}\in \left\{12\text{to}24\right\},\forall p,\forall f_{p}$$ (6)

where $x_{p,f_{p}}^{s}$ are discrete numbers for p∈℘, $f_{p}\in {ℱ}_{p}$ and s∈SS.

The distances between stoppers must not be lower than 1 m and the distances between saws must not be lower than 6 m.


$$\left|x_{p,f_{p}}^{s_{1}}-x_{p,f_{p}}^{s_{2}}\right|\geq 1,\forall p,\forall f_{p},\forall s_{1},s_{2}\in \left\{\text{S}1,\text{S}2,\text{S}3\right\},s_{1}\neq s_{2}$$ (7)

$$\left|x_{p,f_{p}}^{s_{1}}-x_{p,f_{p}}^{s_{2}}\right|\geq 6,\forall p,\forall f_{p},\forall s_{1},s_{2}\in \left\{\text{FS},\text{MS}1,\text{MS}2\right\},s_{1}\neq s_{2}$$ (8)

For the first feed, the jagged end is cut using the FS without stoppers; otherwise, a stopper is engaged. For the last feed, the other jagged end is cut using the FS without a movable saw. At least one saw is engaged with every feed.


$$\sum e_{p,f_{p}}^{s}= \{\begin{array}{c} 0,f_{p}=1\\ 1,otherwise \end{array},\forall p,\forall f_{p},\forall s\in \left\{\text{S}1,\text{S}2,\text{S}3\right\}$$ (9)

$$\sum e_{p,F_{p}}^{s}=0,\forall p,\forall s\in \left\{\text{MS}1,\text{MS}2\right\}$$ (10)

$$\sum e_{p,f_{p}}^{s}\geq 1,\forall p,\forall f_{p},\forall s\in \left\{\text{FS},\text{MS}1,\text{MS}2\right\}$$ (11)

The set of positions of the engaged stoppers and saws in any feed of any pattern is denoted by


$${\text{ES}}_{p,f_{p}}=\left\{x_{p,f_{p}}^{\text{s}};\forall s\in \text{SS },e_{p,f_{p}}^{s}\neq 0\right\}=\left\{ES_{p,f_{p}}^{1},ES_{p,f_{p}}^{2},\ldots \right\},\forall p,\forall f_{p}$$ (12)

where


$$ES_{p,f_{p}}^{i}<ES_{p,f_{p}}^{i+1},\forall p,\forall f_{p},\forall i$$ (13)

No stopper is used for the first feed of any pattern. For the other feeds in a pattern, only one stopper is engaged. The length of Cut 1 is represented as zero for the jagged end. The lengths of all the cuts are the distances between two contiguous engaging stopper or saws. When a few saws are disengaged during cutting, the machine produces only one or two cuts. The cut length value of zero represents that the product is not made in the cut of corresponding feed and pattern.


$$l_{p,1}^{c}= \{\begin{array}{c} ES_{p,1}^{c}-ES_{p,1}^{c-1},1<c\leq {\mathrm{ℰS}}_{p,1}\\ 0,otherwise \end{array},\forall p,\forall c$$ (14)

$$l_{p,f_{p}}^{c}= \{\begin{array}{c} ES_{p,f_{p}}^{c+1}-ES_{p,f_{p}}^{c},1\leq c<{\mathrm{ℰS}}_{p,f_{p}}\\ 0,otherwise \end{array},\forall p,\forall f_{p}\left[f_{p}\neq 1\right],\forall c$$ (15)

Here, ∥⋅∥ is the cardinality of the set and $l_{p,f_{p}}^{c}=0$ represents that either the cut *c* of feed *f*_*p*_ of pattern *p* is the jagged end or there is an absence of engaging saws.

The total length of any cutting pattern must not exceed the length of the ARL chosen for the pattern, and the product length of each cut must be restricted by customer orders.


$$\sum_{f_{p}\in {ℱ}_{p}}\sum_{c=1}^{3}l_{p,f_{p}}^{c}\leq ARL_{p},\forall p$$ (16)

$$l_{p,f_{p}}^{c}\in \left\{0,PL_{1},PL_{2},\ldots ,PL_{L}\right\},\forall p,\forall f_{p},\forall c$$ (17)

Variables $m_{p,f_{p},c}^{l}$ are used to indicate when *l* is produced by any pattern, feed or cut.


$$m_{p,f_{p},c}^{l}= \{\begin{array}{c} 1,l_{p,f_{p}}^{c}=PL_{l}\\ 0,l_{p,f_{p}}^{c}\neq PL_{l} \end{array},\forall l,\forall p,\forall f_{p},\forall c$$ (18)

The variable $q_{l}$ accumulates *l* produced by any pattern, feed or cut. It represents the product of the quantity of cutting pattern $q_{p}$ and the total number of products length *l* in pattern *p*. The total number of products length *l* produced from any pattern must satisfy the total demand of customers.


$$q_{l}=\sum_{p\in ℘}\left(q_{p}\cdot \left(\sum_{f_{p}\in {ℱ}_{p}}\sum_{c=1}^{3}m_{p,f_{p},c}^{l}\right)\right),\forall l$$ (19)

$$q_{l}\geq Q_{l},\forall l$$ (20)

The cutting time depends on the saw position between feeds and is 55 s in case of no changes and 600 s otherwise. Define $t_{p,1}^{c}=55$ for $c\in \left\{1,2,3\right\}$, where p∈P. For other feeds of any pattern, we have


$$t_{p,f_{p}}^{c}= \{\begin{array}{c} 55,x_{p,f_{p}}^{s}=x_{p,f_{p}-1}^{s}\\ 600,x_{p,f_{p}}^{s}\neq x_{p,f_{p}-1}^{s} \end{array},\forall s,\forall p,\forall f_{p}\left[f_{p}\neq 1\right]$$ (21)

## Solution Approach

As briefly mentioned in the introduction section, APOA is proposed to cope with real-world CSPs. To deploy APOA effectively, there are certain procedures needed to be carried out prior to APOA execution. First, using the available ARLs and customer orders as inputs, a cutting pattern is generated for which stopper/saw positions and feed sequences must be specified. To save machine setup time due to frequent change of cutting patterns, each pattern must fulfill at least one size of the final products ordered. The pattern must also satisfy the constraints in the mathematical model to ensure its manufacturability. Then the number of ARLs needed, the final products produced so-far, and the total trim loss are calculated. The final products obtained after applying this cutting pattern are subtracted from the total customer orders, and the remaining final products that are still needed to be cut are recalculated. (More explanation can be founded in *Cutting Pattern Generation*). Next, only the remaining final products are considered in generating the next cutting pattern. It is noted that every time a cutting pattern is employed, the remaining final products decrease. This characteristic can be considered as a network problem where the vertices are the states of the remining final products and the edges are the number of repeats of each cutting pattern. This network representation is discussed in *Problem Space Structuring*.

Each cutting pattern employed implies a progress toward completion of the total orders. In the network term, this means an advancement to reach the final vertex. However, each pattern may yield a different objective function value. Moreover, there could be a large number of possible patterns that can be generated. *Problem Space Structuring* was designed so that the solution search was quick and effective, while the cost was being minimized without loss in productivity. This is done through the rules set in *Suboptimal Solution Elimination* and the allowable threshold in *Manufacturer Loss Limit*.

**Fig 2:**
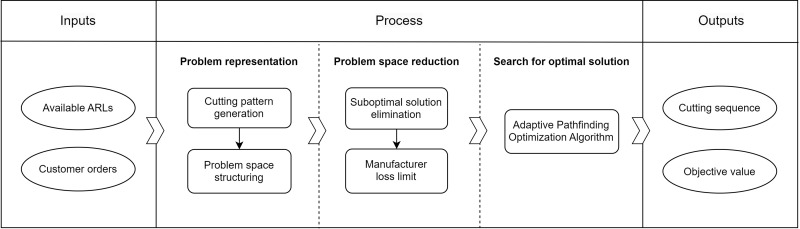
Block diagram illustrating the solution approach.

Lastly, to solve the CSP that satisfies any customer order under the machine and resource constraints, APOA is devised. APOA is an adaptive pathfinding optimization algorithm. It combines WACO (a variant of ACO) with Dijkstra’s algorithm. The adaptability is in its dynamic employment of the search strategy to balance efficiency and scalability. For smaller problems, Dijkstra’s algorithm is utilized; but for larger ones, WACO is employed. The outputs of this process are cutting sequences along with the objective value, ensuring that customer orders are fulfilled optimally. The efficiency of obtaining optimal solutions depends on the size of the search space, with the chances of finding the best solution increasing as the problem space is reduced. The entire procedure above is summarized in Fig 2.

### Problem representation

This section discusses the configurations of the cutting machine, focusing on elements such as stoppers and cutting saws. Subsequently, the process of generating the cutting patterns is elaborated. Finally, the problem representation is described and assessed for the complexity.

#### Cutting pattern generation.

Each cutting pattern begins with the first feed. Subsequently, the machine advances the pre-cut bar toward an engaging stopper and executes the necessary cuts in each feed, resulting in various final products. The process continues until the last feed of the pattern is reached, in which the remaining pre-cut bar is moved to the designated stopper and cut by the FS. All the final products are transferred to the two operating cooling beds at the end of the line. The repetitive feeds between the initial and final feeds are known as intermediate feeds. Furthermore, the positions of the stoppers on the FS and those between the saws determine the lengths of the final products. To eliminate any further processing, the stopper– and saw–FS distances should coincide with the lengths of the final products.

Each of the two cooling beds can accommodate only one length of the final product; hence, the number of product lengths in a cutting pattern is limited to two. Multiple cutting patterns are required for more than two product lengths in an order, and any combination of the two product lengths can be selected to generate a cutting pattern. These lengths are denoted as x and y, where x >  y. If a pattern contains a single product length, then x =  y =  0. The three-saw cutting machine can cut up to three different lengths of the final products in a pattern. Hence, assuming that the saw and stopper positions remain unchanged, a maximum of two lengths can be exercised owing to the cooling bed restriction.

The two-length restriction must also be enforced by the stopper positions, i.e., only two out of the three stoppers can be used in a cutting pattern. Let LS1 and LS2 be logical positions of the two stoppers, where LS1 is -17 to -6 meters away from the FS with an increment of 0.1 m. Similarly, LS2 is -18 to -7 meters away with the same increment. In this configuration, LS2 must always be to the left of LS, and thus, LS2 must have a more negative value compared to LS1. This is because LS2 falls within the range of the values of S3 and S2, whereas LS1 covers those of S2 and S1.

The positions of the stoppers and cutting saws can be denoted by a 5-tuple representing the relative positions of LS2, LS1, FS, MS1 and MS2. The position of FS is set as the reference point; hence, it is always equal to zero. The values of LS2 and LS1 are negative, indicating that they are situated to the left of the FS. Contrastingly, the positive values of MS1 and MS2 indicate that their positions are to the right of the FS. Table 1 lists the feasible positions for different values of (x, y). N/A represents the case without any value, which occurs when no stopper or MSs are engaged in cutting.

**Table 1.** Feasible stopper/saw positions.

x	y	Feasible positions (LS2, LS1, FS, MS1, MS2)	Remark
9	6	(-9, -6, 0, 9, 18) (-9, -6, 0, 6, 12) (-9, -6, 0, 9, 15) (-9, -6, 0, 6, 15)	–
6	0	(N/A, -6, 0, 6, 12)	S2 is disengaged.
14	12	(-14, -12, 0, 12, 24) (-14, -12, 0, 14, N/A)	Maximum length of MS2 is 24 m. Consequently, if MS1 is used to cut a length of *x* = 14 m, then MS2 cannot be employed to cut a length *y* = 12 m.
8.5	8	(N/A, -8, 0, 8.5, 17) (N/A, -8, 0, 8.5, 16.5) (N/A, -8, 0, 8, 16.5) (N/A, -8.5, 0, 8.5, 16.5) (N/A, -8.5, 0, 8, 16.5) (N/A, -8.5, 0, 8, 16)	Owing to the machine constraint, any stopper must be positioned at least a meter away from the other stoppers. If LS1 is set at -8 m, LS2 could not be set at -8.5 m; hence, LS2 must not engage.

**Fig 3:**
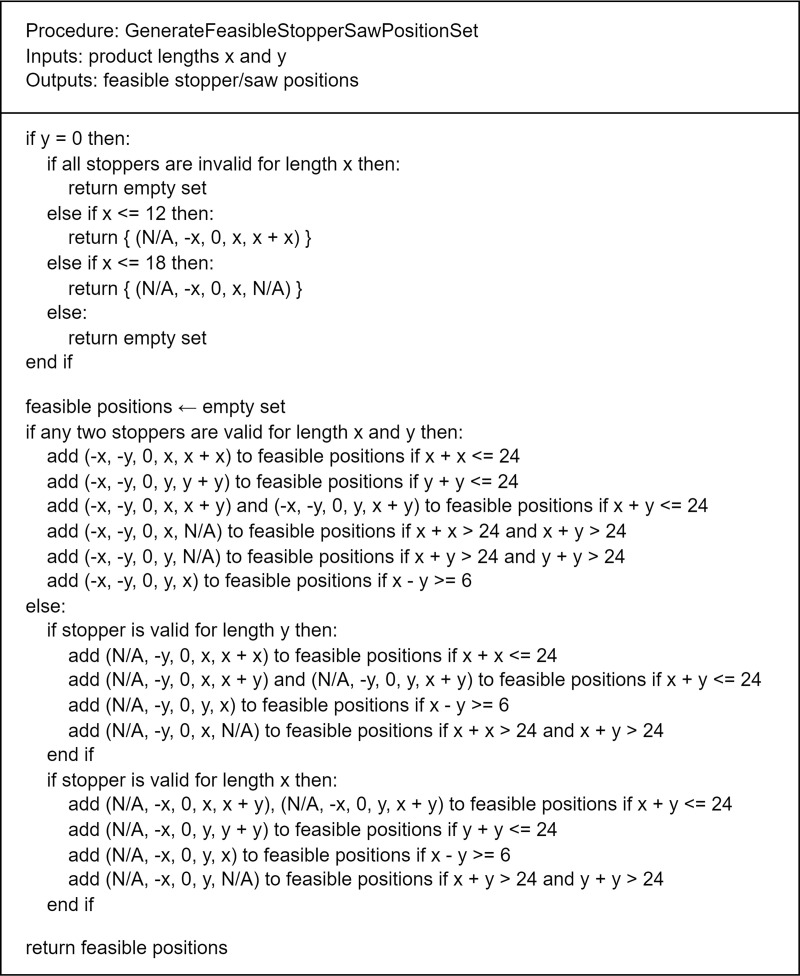
Pseudocode to generate feasible stopper/saw positions.

A set of feasible positions (LS2, LS1, FS, MS1 and MS2) is generated using the pseudocode shown in Fig 3. Notably, this pseudocode is tailored to adhere strictly to the constraints of the current machine, and it should be customized for different machines.

In addition to the positions of cutting saws and stoppers, their engagement (E) and disengagement (D) statuses must be specified. Different conditions are applied to different feeds of the cutting pattern. For the first feed, all stoppers are disengaged, but the FS is engaged. Optionally, MS1, MS2 or both can be engaged. Only one stopper and at least one saw are used for each intermediate feed. For the last feed, only one stopper and FS are engaged, whereas the two movable saws are disengaged. Owing to these constraints, each feed has a corresponding stopper/saw status. Table 2 lists all possible combinations of stopper/saw statuses.

**Table 2.** Statuses of (LS2, LS1, FS, MS1, MS2) for different feeds.

Feed	Corresponding stopper/saw statuses
First feed	(D,D,E,E,E), (D,D,E,E,D), (D,D,E,D,E)
Intermediate feed	(D,E,E,D,D), (D,E,D,E,D), (D,E,D,D,E), (D,E,E,E,D), (D,E,E,D,E), (D,E,D,E,E), (D,E,E,E,E), (E,D,E,D,D), (E,D,D,E,D), (E,D,D,D,E), (E,D,E,E,D), (E,D,E,D,E), (E,D,D,E,E), (E,D,E,E,E)
Last feed	(D,E,E,D,D), (E,D,E,D,D)

However, for x and y, there is a potential inclusion of invalid cut lengths — those that are neither x nor y — which are eliminated to uphold feasibility. Table 3 provides examples of cut lengths when (x, y) =  (9 m, 8.5 m) and stopper/saw positions are (N/A, -8.5, 0, 9, 18). An invalid stopper occurs when the position of the engaged stopper violates the machine constraint. Specifically, the position of the engaged stopper is too close to another stopper.

**Table 3.** Cut lengths when (x, y) =  (9 m, 8.5 m) and the stopper/saw positions are (N/A, -8.5, 0, 9, 18).

Feed	All possible stopper/saw statuses	Cut lengths
First feed	(D,D,E,E,E) (D,D,E,E,D) (D,D,E,D,E)	[0, 9, 9] [0, 9, 0] Invalid cut length
Intermediate feed	(D,E,E,D,D) (D,E,D,E,D) (D,E,D,D,E) (D,E,E,E,D) (D,E,E,D,E) (D,E,D,E,E) (D,E,E,E,E) (E,D,E,D,D) (E,D,D,E,D) (E,D,D,D,E) (E,D,E,E,D) (E,D,E,D,E) (E,D,D,E,E) (E,D,E,E,E)	[8.5, 0, 0] invalid cut length invalid cut length [8.5, 9, 0] Invalid cut length invalid cut length [8.5, 9, 9] invalid stopper invalid stopper invalid stopper invalid stopper invalid stopper invalid stopper invalid stopper
Last feed	(D,E,E,D,D) (E,D,E,D,D)	[8.5, 0, 0] invalid stopper

**Fig 4:**
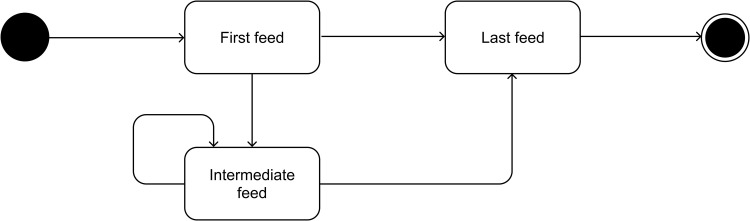
State diagram of the sequence in a cutting pattern.

The cutting process commences with the first feed, progresses through a series of intermediate feeds and ends with the final feed. In some patterns, the last feed may directly follow the first. Fig 4 shows the sequence of feeds constituting a cutting pattern. The total number of final products obtained from the pattern can be tallied from the cut bars with lengths x and y. The trim loss can be calculated by comparing the collective total lengths of these cut bars with the length of the ARL bar used to cut them.

Computerized cutting-pattern generation commences by specifying the ARL bar lengths x and y. Initially, feasible positions for stoppers and saws are generated, as outlined in the pseudocode in Fig 3. Subsequently, valid or feasible feeds are generated according to the rules defined in the state diagram shown in Fig 4. These feeds consider the feed types (first, intermediate or last feed) and the corresponding statuses (engagement or disengagement) of the stoppers and saws. Finally, a sequence of feeds is generated using the ARL bar length as a constraint. The systematic process of generating the cutting patterns can be delineated as follows: (ARL, x, y) - > (stopper/saw positions) - > (stopper/saw statuses) - > (feeds) - > (patterns). Table 4 provides examples of patterns generated by ARLs, cut lengths and stopper/saw positions.

**Table 4:** Patterns generated by ARLs, cut lengths and stopper/saw positions.

#	ARL	Cutting patterns	Products	Trim length
1	36	[0, 9, 9] ([8.5, 0, 0]) [8.5, 0, 0]	9Mx2 + 8.5Mx2	1
2		[0, 9, 0] ([8.5, 9, 0]) [8.5, 0, 0]	9Mx2 + 8.5Mx2	1
3		[0, 9, 0] ([8.5, 0, 0], [8.5, 0, 0]) [8.5, 0, 0]	9Mx1 + 8.5Mx3	1.5
4	38	[0, 9, 9] ([8.5, 0, 0]) [8.5, 0, 0]	9Mx2 + 8.5Mx2	3
5		[0, 9, 0] ([8.5, 9, 0]) [8.5, 0, 0]	9Mx2 + 8.5Mx2	3
6		[0, 9, 0] ([8.5, 0, 0], [8.5, 0, 0]) [8.5, 0, 0]]	9Mx1 + 8.5Mx3	3.5
7	45	[0, 9, 9] ([8.5, 9, 0]) [8.5, 0, 0]	9Mx3 + 8.5Mx2	1
8		[0, 9, 9] ([8.5, 0, 0], [8.5, 0, 0]) [8.5, 0, 0]	9Mx2 + 8.5Mx3	1.5
9		[0, 9, 0] ([8.5, 9, 9]) [8.5, 0, 0]	9Mx3 + 8.5Mx2	1
10		[0, 9, 0] ([8.5, 9, 0], [8.5, 0, 0]) [8.5, 0, 0]	9Mx2 + 8.5Mx3	1.5
11		[0, 9, 0] ([8.5, 0, 0], [8.5, 9, 0]) [8.5, 0, 0]	9Mx2 + 8.5Mx3	1.5
12		[0, 9, 0] ([8.5, 0, 0], [8.5, 0, 0], [8.5, 0, 0]) [8.5, 0, 0]	9Mx1 + 8.5Mx4	2

#### Problem space structuring.

**Fig 5:**
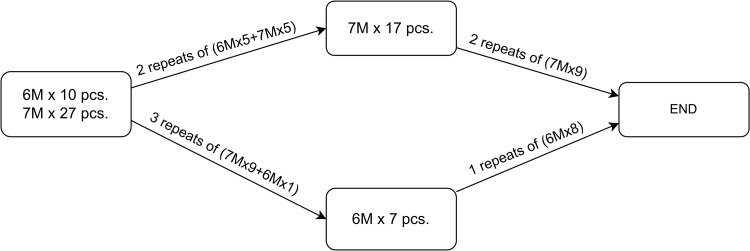
Example of graph representation of an CSP.

Fig 5 shows a directed acyclic graph, which is a representation of CSP, wherein each vertex signifies a cutting state representing the remaining products that need to be sequentially processed. Each edge of the graph corresponds to a cutting pattern along with the number of repetitions starting from one and increasing until at least one final product (with x, or x and y lengths) in the order is fulfilled. This ensures that at least one length of the remaining order is satisfied at any particular state besides the beginning state (the leftmost state in the figure), i.e., the state transitions represent instances in the cutting plan that completely satisfy at least one product length.

The solution to the CSP in this graph representation is the path from the starting state to the ending state. The starting state comprises all the product lengths and their ordered quantities. The ending state can be a state with an empty remaining order (all ordered products are satisfied) or zero edges for further transition. The aforementioned path comprises states and edges representing the fulfilment of order by the cutting patterns and their repetitions.

Practically, once the demand is consolidated, the total quantities of the final products ordered must be checked against those available in the inventory and the remaining unsatisfactory amounts are processed. These become the initial state of the graph representation. Subsequently, cutting patterns are generated based on all combinations of the one or two lengths in the remaining order and available ARL bars.

The number of possible complete paths from the start to end vertices determines the problem size, which can be exhaustively quantified by considering all the possibilities of vertex transitions. To estimate the number of paths in relation to the problem inputs, we first define a set of product lengths L with cardinality $\left|ℒ\right|$ =  *L*. Let the *outdegree* of a vertex be the number of outgoing edges that can entirely satisfy at least one product length in the order. For simplicity, let us assume that all edges satisfy a single product length. Thus, the outdegree of a vertex with the remaining product lengths L to be fulfilled is the permutation of the elements in L. Let the $i^{th}$ permutation be denoted as $\left(a_{i1},a_{i2},\ldots ,a_{iL}\right)$, where $a_{il}$ is the product length *l* of permutation *i*. Because the ARL bar should be longer than the product, a set of patterns ${℘}_{a_{ij}}$ exists corresponding to any product length $a_{il}$.

For instance, given a product length of 9 m and ARL length of 36 m, there exist numerous patterns, including (9Mx2, 8.5Mx2), (9Mx1, 8.5Mx3), or (9Mx4). The number of possible ways to select the sequence of patterns of the $i^{th}$ permutation is the product of the size of ${℘}_{a_{il}}$ for l=1,2,…,L. Therefore, the problem space size or the total number of paths for each permutation is derived using the following equation:


$$Problemspacesize=\sum_{i=1}^{L!}\left(\prod_{l=1}^{\text{L}}\left|{℘}_{a_{il}}\right|\right)$$ (22)

where ${℘}_{a_{ij}}\subseteq ℘$ is a set of patterns that can accommodate the length $a_{ij}$ and does not produce the product of length $\left\{a_{i1},a_{i2},\ldots ,a_{i\left(j-1\right)}\right\},$ and$\left|�\right|$represents the cardinality of a set.

The number of paths is related to *L* and $\left|{℘}_{a_{ij}}\right|$, where $\left|{℘}_{a_{ij}}\right|$ denotes the number of patterns in a particular cutting state. Because the number of cutting patterns does not depend on the inputs *L*, it is erroneous to associate the number of paths solely to inputs, such as L or ARL bars, as the combination of product lengths in L and the number of ARL lengths produce different sets of patterns.

The problem space size aligns more closely with the patterns than inputs. To address the problem space size in terms of the input sizes, the best- and average-case scenarios are considered. For the best-case scenario, only one path from the start to the end state exists for each permutation, yielding the complexity value of L!. The term ‘best’ signifies the smallest possible problem space. In the average-case scenario, we consider that each length $a_{il}$ can exhibit multiple patterns. The multiplication of *L* terms of different bounded numbers, particularly $\left(\prod_{l=1}^{\text{L}}\left|{℘}_{a_{il}}\right|\right)$ in Eq. (22), can be estimated as $b^{L}$, where $1<b<\left|\text{℘ }\right|$ and *b* varies based on the problem instance. This facilitates the estimation of the problem space size.


$$Problemspacesize\approx b^{L}L!$$ (23)

Eq. (23) indicates that the problem space size is associated with the variable *b* and the number of product lengths. Power term ($b^{L}$) and factorial term (L!) indicate that the problem space complexity increases significantly when *L* increases, even with a small increment in *L*.

### Problem space reduction

The complexity of the problem space, characterized by power and factorial terms, creates a significant computational burden. This challenge can be mitigated through problem space reduction techniques. During the graph construction process, the number of feasible edges at each vertex contributes to a substantial expansion of the graph. By reducing the number of feasible edges, both the number of vertices and edges in the problem graph are decreased, leading to a smaller search space. Mathematically, this reduction is equivalent to lowering the base *b* in the power term of problem space size. To achieve this, we introduced two problem space reduction methods: Suboptimal Solution Elimination and Manufacturer Loss Limit.

#### Suboptimal solution elimination.

Suboptimal solution elimination leverages the concept of the dominance rule [31] to discard edge options that lead to suboptimal solutions. Two key dominance rules are applied: Identical Product Set and Identical Subsequent Vertex.

**Rule 1: Identical Product Set** – If multiple feasible patterns generate the same product set, only the pattern that minimizes both cutting time and trim loss is retained.

After the Cutting Pattern Generation process, applying the Identical Product Set rule filters out dominated patterns, leaving a set of non-dominated patterns that yield the same product set but with the best combination of cutting time and trim loss. For instance, in Table 4, patterns #1, #2, #4, and #5 produce the same product set, but only the pattern with the lowest cutting trim loss is kept. Similarly, different patterns generated from the same ARL bar can result in the same product set. For example, patterns #10 and #11 use different orders of intermediate feeds but ultimately generate the same product set. If multiple patterns share the lowest cutting time and trim loss, one is chosen arbitrarily. This filtered set of non-dominated patterns is referred to as the feasible pattern set.

**Rule 2: Identical Subsequent Vertex** – If multiple edges lead to the same subsequent vertex, the edge with the lowest cost—determined by a predefined weighted cost function—is retained.

At a particular expanding vertex, multiple edges (patterns with different numbers of repetitions) may lead to the same subsequent vertex (i.e., the same set of remaining orders). For instance, if the remaining order for a cutting state is 9Mx10 and 6Mx15, one edge may produce 9Mx10 +  6Mx2 from two iterations of 9Mx5 +  6Mx1 on a 52-meter ARL bar, resulting in the state 6Mx13 with a total trim loss of 2 meters (1 meter per repeat). Alternatively, another edge may yield 9Mx12 +  6Mx2 from two iterations of 9Mx6 +  6Mx1 on a 60-meter ARL bar, leading to the same subsequent vertex but with over-rolls of 9Mx2. Although the latter option incurs over-rolling, the former produces a trim loss. To determine the most suitable pattern and repetition for a given vertex, the weighted cost of each edge is computed, and the edge with the lowest cost is selected.

#### Manufacturer loss limit.

The Manufacturer Loss Limit method, inspired by practical manufacturing constraints, reduces the number of edge options during graph expansion by applying a trim loss threshold to the feasible patterns. Only patterns that produce a trim loss within the specified limit, or patterns that generate a single product length, are retained.

Manufacturers aim to balance minimizing trim loss while maximizing yield during steel cutting. To achieve this, we introduce a trim-loss limit that represents the maximum allowable trim loss for each ARL bar. Based on the size of the trim collection bin and industry standards, a trim-loss limit of 2–4 meters is recommended. However, a potential drawback of this method is that if all patterns corresponding to a specific product length $PL_{l}$ are pruned, it becomes impossible to fulfill orders for that product length. To prevent this, cutting patterns that produce a single product length are always retained, ensuring that all final product lengths can be cut.

### Search for the optimal solution

In the graph representation, the optimal solution corresponds to the shortest path, defined by the sequence of cutting steps from the starting node to the ending node. The path’s distance reflects the total weighted cost. To address this, we propose the APOA, which combines the newly introduced WACO, a variant of the ACO algorithm, with the conventional shortest-path algorithm, Dijkstra’s algorithm.

#### Adaptive pathfinding optimization algorithm.

The APOA dynamically adjusts its strategy based upon the problem’s size and complexity. For smaller problem sets, Dijkstra’s algorithm [32] are efficient and guarantee optimality. For larger problems, where time and hardware constraints are significant, WACO is employed to provide near-optimal solutions. By integrating these methods, APOA balances efficiency and scalability, handling problems of various sizes.

The proposed method uses a lazy loading technique [33] to manage memory efficiently. Vertex and edge data are generated only as needed, reducing memory consumption. The graph is not fully expanded, so memory uses is optimized during the search for the shortest path. Initially, the algorithm uses Dijkstra’s algorithm, which either completes successfully or is stopped early based on a predetermined stopping criterion, such as the number of expanded vertices. If the algorithm is stopped early, APOA switches to WACO to continue the search.

#### Wandering ant colony optimization algorithm.

**Fig 6:**
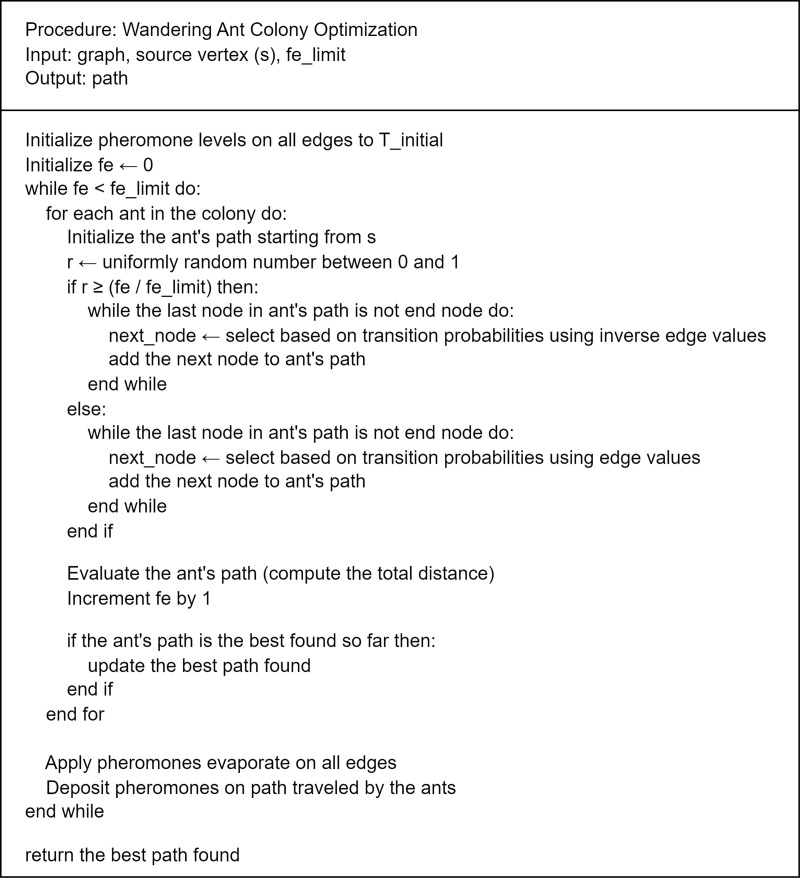
Pseudocode of the WACO algorithm.

The WACO algorithm addresses limitations related to time and hardware by iteratively refine near-optimal paths in large graphs. Unlike deterministic algorithms, WACO is stochastic, enhancing known paths while exploring new ones. Based on ACO principles, WACO mimics ant foraging behavior, where virtual ants traverse the graph and deposit pheromone trails on edges depending upon the path quality (related to the objective function value). The pseudo code of the WACO algorithm is given in Fig 6.

A key distinction in WACO is the introduction of wandering ants, which explore new paths instead of following established pheromone trails. This mitigates the issue of premature convergence found in conventional ACO where pheromone trail becomes concentrated on certain paths, ensuring broader search space exploration. The balance between exploitation (following pheromone trails) and exploration (wandering) is central to WACO’s effectiveness.

Initially, WACO prioritizes wandering to explore the graph broadly, for instance, at 20% algorithm progression, ants have a 20% chance of following pheromones and an 80% chance of wandering. A random number between 0 and 1 is compared to the progression ratio to determine the ant behavior. As the algorithm advances, the probability of wandering decreases. By the final stages, wandering probability drops close to 0%, and ants fully depend on edge values derived from pheromone and heuristic factors. This process continues until the function evaluation (*fe*) reaches the limit, where the algorithm concludes.

#### Conditions governing the use of WACO and Dijkstra’s.

The APOA selects the appropriate algorithm based on the graph’s size, complexity and time constraints. For smaller or simpler graphs, Dijkstra’s algorithm is employed, as it guarantees the optimal solution efficiently through systematic exploration of all vertices and edges. Its deterministic nature ensures precision when computation time is not a limiting factor.

For large-scale or time-sensitive problems, APOA switches to WACO. WACO’s flexibility allows adjustment of the number of function evaluations limit (*fe_limit*) to explore the solution space and return near-optimal solutions more quickly. Its wandering ants prevent premature convergence, ensuring comprehensive search space exploration. This capability is particularly valuable for large problems where Dijkstra’s exhaustive graph expansion would be impractical.

By employing Dijkstra’s algorithm for exact solutions and WACO for rapid, near-optimal solutions, APOA adapts to each problem’s unique constraints. This ensures the most effective solution based on the task’s specific demands.

## Results and discussion

Seven product groups (PG-1 to PG-7) are provided from the manufacturer’s dataset, sorted by problem size in ascending order. Each product group (PG) corresponds to a set of inputs, which include available ARL bars and aggregated orders, grouped by steel grade and cross-sectional area from multiple customers. The tests are conducted on these product groups to evaluate and performance of the proposed optimization approach across varying problem sizes. The current hardware is an Apple M2 processor with 24 GB of memory.

Table 5 summarizes the number of different input lengths in the corresponding product group and problem space in the number of vertices, edges and paths. For instance, the graph representation of the product group PG-5 has approximately one million vertices, 30 million edges, and more than 25,000 million paths for eight different order lengths with six different ARL bar lengths. The exact number of vertices and paths for PG-6 and PG-7 cannot be obtained with the current hardware due to memory limit.

**Table 5:** Product group input lengths and the corresponding problem sizes.

Product Group	Number of distinct lengths		Problem sizes					
			Not applied trim loss limit			Apply trim loss limit = 2 meters		
	ARL	Product	Vertex	Edge	Path	Vertex	Edge	Path
PG-1	13	4	9 K	52 K	83 K	7 K	41 K	64 K
PG-2	9	5	10 K	208 K	4 M	5 K	74 K	783 K
PG-3	9	7	297 K	6 M	3,044 M	144 K	2 M	388 M
PG-4	5	7	459 K	12 M	8,208 M	162 K	2 M	471 M
PG-5	6	8	989 K	30 M	25,108 M	306 K	5 M	969 M
PG-6	9	12	Unobtainable			Unobtainable		
PG-7	11	18	Unobtainable			Unobtainable		

### Test of convergence

Comparative tests of convergence were performed to ensure that WACO can potentially discover good solutions compared to other random search algorithms. The medium-sized datasets PG-3 and PG-4 were selected for comparison because they are relatively large problems with computationally solvable global optimal. The competing algorithms for path searching in graph are conventional ACO, WACO, and random key-based GA (rkGA), which is a variant of the genetic algorithm [34]. ACO conceptually balances the exploration–exploitation strategy. The ants communicate through a pheromone trail, and a predefined heuristic promotes exploitation, and the pheromone evaporation mechanism enhances exploration. The rkGA, a genetic algorithm tailored to address the shortest path routing problem, employs random key encoding analogous to our problem.

The individual iterations of each algorithm are limited by the same number of function evaluations. Except for the ant behavior, ACO and WACO have the same configuration. The hyperparameters for the ACO and WACO are the pheromone evaporation rate and alpha and beta in the edge value calculation. The hyperparameters for rkGA are the crossover, mutation and immigration rates established by hyperparameter tuning [35]. In the experiment, 30 runs of each algorithm were averaged to avoid bias from the best- or worst-case scenarios, which may occur in some iterations because of randomness.

**Fig 7:**
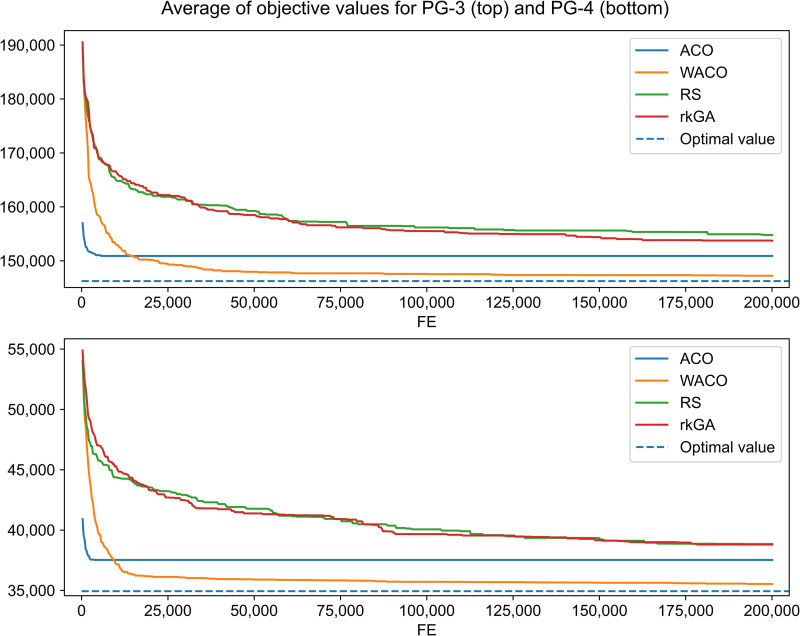
Convergence graph of various random-based search approaches.

Fig 7 illustrates the convergence of three random search algorithms. The horizontal axis tracks function evaluations, while the vertical axis represents the average objective values of the best solutions across iterations. The global optimum is marked by the dashed line, and the *fe_limit* is set to 200,000 for all runs. The rkGA algorithm steadily reduces objective values as *fe* increases but reaches the optimal value more slowly than ACO and WACO. ACO quickly approaches the local optimum but stagnates without further improvement. In contrast, WACO not only finds good solutions rapidly but continues to refine them towards a near-optimal value. WACO achieves the best final average objective function among the three algorithms. Random search (RS) results are also included as a benchmark baseline.

These results demonstrate that WACO effectively balances exploration and exploitation to obtain superior solutions in a Directed Acyclic Graph (DAG). The lines show the objective values converging toward optimal solutions during function evaluations. Compared to other algorithms, WACO achieves the lowest average objective values with fewer function evaluations. By enabling ants to wander and explore new paths, WACO can discover superior solutions or paths that might be overlooked if all ants strictly followed pheromone trails, as in conventional ACO.

### Cutting plan and performance

**Fig 8:**
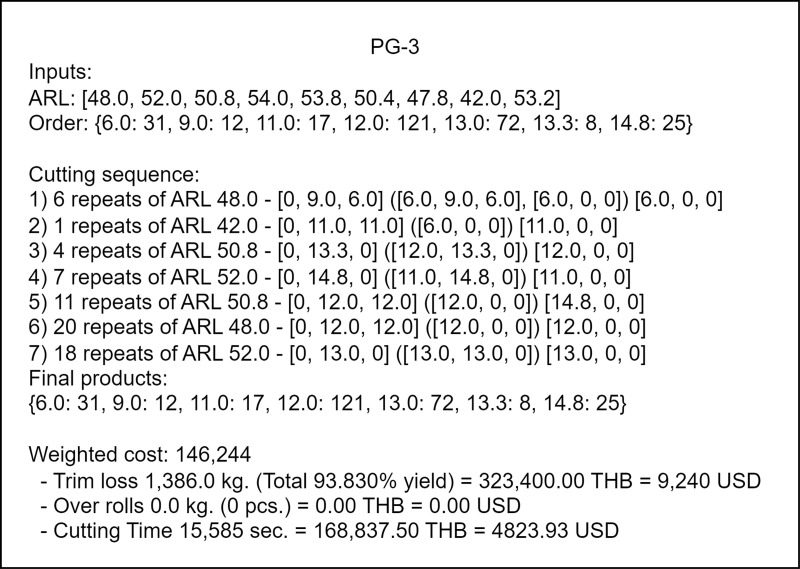
Example of a cutting plan and weighted cost report.

The cutting plan is a recipe for the manufacturer staff. As shown in Fig 8, it composes of the information on the available ARL bars, aggregated orders, a sequence of cutting patterns and their repetitions, total pieces of final products, and the cost report. For each step in the sequence, the following three types of information are provided: (1) number of pattern repetitions, (2) ARL bars used for a specific pattern and (3) sequence of feeds containing the first, intermediate and final feeds. The number of pattern repetitions must ensure that at least one length of the final product is obtained.

For example, the number of distinct ARL bar lengths and final product (or order) lengths of PG-3 in Table 5 are nine and seven, respectively. The details of these lengths as well as the number of pieces of the final products needed are shown at the top of Fig 8. The algorithm uses this information to generate the solution. This solution specifies the number of different ARL bars needed, cutting patterns, and the total final products obtained after executing these cutting patterns. The manufacturer uses the outputs of this solution as the cutting plan for the corresponding product group. The first feed of the first cutting pattern provides one 9-m and one 6-m long final products. In the second feed of this first cutting pattern, two 6-m and one 9-m long final products are cut. In the third and final feeds, only one 6-m long product is cut in each of the feed. Two 9-m and five 6-m long final products are obtained using four feeds in this pattern. This pattern repeats six times, and the subsequent cutting patterns follow the same process until completion. The total final products are aggregated from the products of all patterns.

The cost report comprises trim loss (with yield percentage), over rolls, cutting time, and weighted cost. Trim loss, over rolls, and cutting time are determined using equations (1) – (3). The weighted cost is calculated by incorporating these factors along with the given importance weights as detailed in equation (4). The yield percentage was computed by considering the difference in the weight of the original steel bloom and the total weight loss, which comprised the weights from the trim and material losses at the two jagged ends. This difference was subtracted from the weight of the original bloom, and the remaining value was used to calculate the yield as a percentage (% yield). A higher percentage value is preferable and a cutting plan with a high % yield and a low number of over rolls is desirable.

Table 6 lists the performance results for the seven product groups (PG-1–PG-7), where key objectives: trim loss, over rolls, and cutting time are assigned importance weights of 0.4, 0.5, and 0.1, respectively. Each column in the table represents a comparison between different algorithms. For each cutting plan, the yield percentage, over rolls, and cutting time are calculated, with lower objective values being preferred. A computation time limit of one hour was set based on an agreement with the manufacturer. The bolded values in the table represent the best solutions compared to other methods for the same product group.

**Table 6:** Comparison of the cutting plans by Dijkstra’s, WACO, ACO, rkGa, and APOA algorithms.

Subject	Dijsktra’s	WACO	ACO	rkGA	APOA
PG-1					
Yield (%)	98.94	98.94	98.94	98.94	98.94
Over rolls (pcs.)	6	6	6	6	6
Cutting time (sec.)	234,995	234,995	234,995	234,995	234,995
Objective value (no unit)	**384,928**	**384,928**	**384,928**	**384,928**	**384,928**
Computational time (sec.)	**18**	55	43	43	**18**
PG-2					
Yield (%)	96.71	96.71	96.69	96.71	96.71
Over rolls (pcs.)	3	3	1	0	3
Cutting time (sec.)	12,020	12,020	12,020	12,460	12,020
Objective value (no unit)	**52,334**	**52,334**	52,539	52,418	**52,334**
Computational time (sec.)	**14**	68	50	48	**14**
PG-3					
Yield (%)	93.83	93.81	93.68	93.81	93.83
Over rolls (pcs.)	0	0	0	0	0
Cutting time (sec.)	15,585	15,585	15,585	15,585	15,585
Objective value (no unit)	**146,244**	146,804	149,604	146,804	**146,244**
Computational time (sec.)	**64**	86	64	196	**64**
PG-4					
Yield (%)	96.55	96.55	96.26	96.47	96.55
Over rolls (pcs.)	0	0	0	0	0
Cutting time (sec.)	14,160	14,160	13,715	14,160	14,160
Objective value (no unit)	**34,940**	**34,940**	35,858	35,220	**34,940**
Computational time (sec.)	**49**	94	58	261	**49**
PG-5					
Yield (%)	94.94	94.86	95.17	95.16	94.94
Over rolls (pcs.)	0	0	1	1	0
Cutting time (sec.)	17,950	17,235	16,465	17,785	17,950
Objective value (no unit)	**101,206**	101,831	105,557	105,547	**101,206**
Computational time (sec.)	**112**	200	89	433	**112**
PG-6					
Yield (%)	N/A	97.27	96.74	N/A	97.27
Over rolls (pcs.)	N/A	1	0	N/A	1
Cutting time (sec.)	N/A	45,535	44,215	N/A	45,535
Objective value (no unit)	N/A	**119,551**	126,580	N/A	**119,551**
Computational time (sec.)	N/A	2,718	**711**	N/A	2,718
PG-7					
Yield (%)	N/A	96.22	95.65	N/A	96.22
Over rolls (pcs.)	N/A	3	4	N/A	3
Cutting time (sec.)	N/A	52,105	51,775	N/A	52,105
Objective value (no unit)	N/A	**269,535**	311,122	N/A	**269,535**
Computational time (sec.)	N/A	**2,743**	3,023	N/A	**2,743**

For relatively small product groups PG-1 through PG-5, the solution could be obtained using any of the algorithms tested. However, the optimal solution was consistently achieved by the APOA. The algorithm required less than two minutes of computation time. In contrast, WACO, ACO, and rkGA algorithms generally took slightly longer to compute the solution. Since the APOA and Dijkstra’s pathfinding method guarantee optimality, they consistently provided the best results in terms of both computation time and objective value.

The problem sizes of more complex product groups, PG-6 and PG-7 were calculated from the combinations of ARLs, and final product lengths are unobtainable as previously defined in equation (22) and shown in Table 5. For these product groups, the Dijkstra’s algorithm failed to obtain the final solution due to the extensive size of the graph. Similarly, rkGA could not complete the run because it required a manageable number of vertices for its chromosome encoding, which was not feasible for larger product groups. For these reasons, the notation “N/A” in the table indicates that the solution could not be achieved due to a runtime constraint. On the other hand, WACO and ACO could obtain the final solutions with objective values equals to 119,551 and 126,580, respectively, for PG-6, and 269,535 and 311,122, orderly, for PG-7. It is obvious that WACO could provide better solutions compared to the original ACO.

From Table 6, the results of the APOA approach leverage the strengths of both Dijkstra’s pathfinding and WACO to address a range of problem sizes. For larger problems, WACO is preferable since Dijkstra’s algorithm is less suited for handling complex graphs. Dijkstra’s pathfinding focuses solely on the shortest distance path. It slowly reaches the final vertex because all incumbent paths must be carefully considered to ensure optimality. In contrast, WACO explores paths probabilistically, allowing for faster identification of near-optimal solutions. Rather than relying on deterministic exhaustive searches like Dijkstra’s, WACO uses pheromone trails to guide its search, balancing between exploring new paths and exploiting known good ones. This probabilistic approach allows WACO to manage larger problem spaces more efficiently. The algorithm could provide near-optimal solutions within a tractable time frame, although it does not guarantee optimality. By combining these two methods, the APOA approach ensures that solutions are both efficient and scalable across varying problem complexities.

### Operational cost saving

Table 7 shows the operational cost comparison for product groups PG-1 to PG-7. The table consists of the product groups, product weights, operational costs, and total cost savings. The product weights in tons which represent the total weight of the product ordered by customers. The cost columns provide a comparison between the baseline results from the manufacturer with the results from adaptive pathfinding optimization algorithm. The values are separated into trim loss, over rolls, and total cost. The total cost saving columns shows the savings amount, by weights, and percentage of the baseline cost. Low values for cost columns are desirable. In contrast, higher values for the total cost saving columns indicate significant cost reductions.

**Table 7:** Operational cost comparison and total cost saving.

Product group	Product weight (tons)	Measure	Cost		Total cost saving		
			Baseline (USD)	APOA (USD)	Amount (USD)	By weight (USD/tons)	Percentage (%)
PG-1	4,348	Trim loss	23,780	9,300	14,523	3.34	60.9%
		Over roll	51	9			
		Total	23,831	9,309			
PG-2	410	Trim loss	3,306	2,811	1,831	4.47	39.4%
		Over roll	1,343	6			
		Total	4,649	2,817			
PG-3	702	Trim loss	9,517	9,246	2,366	3.37	20.4%
		Over roll	2,094	0			
		Total	11,611	9,246			
PG-4	194	Trim loss	1,666	1,389	523	2.69	27.3%
		Over roll	246	0			
		Total	1,911	1,389			
PG-5	548	Trim loss	5,771	5,843	10,546	19.24	64.3%
		Over roll	10,617	0			
		Total	16,389	5,843			
PG-6	833	Trim loss	420	377	1,923	2.31	80.2%
		Over roll	1,977	97			
		Total	2,397	474			
PG-7	1,719	Trim loss	2,066	1,531	2,840	1.65	52.4%
		Over roll	3,349	1,043			
		Total	5,414	2,574			

For all product groups (PG-1–PG-7), the total cost saving amounts indicate a positive savings. They are ranging from USD 523 – 10,546 (USD 1 =  THB 35). The lower costs for both trim loss and over rolls are generally achieved by the adaptive pathfinding optimization algorithm. Only PG-5 exhibits a higher trim loss cost with lower over rolls cost. However, this product group shows the highest total cost savings per ton of order (USD 19.24 per ton) compared to baseline performance. This is attributed to high over rolls cost reduction by USD 10,617, whereas the trim loss cost is addition from the baseline result by USD 72.

The cost saving per ton is ranging from USD 1.65 – 19.24 and the cost savings percentage is ranging from 20.4% – 80.2%. To estimate the average values achieved by employing the proposed algorithm, the total cost saving is divided by the total amounts such that the average cost saving per ton of order is (sum of cost savings)/(sum of order weights) =  USD 3.95. On the other hand, the average cost saving percentage is (sum of cost savings)/ (sum of baseline cost) =  52.18%.

## Conclusion

This study investigates the CSP in the context of the steel industry by incorporating cutting conditions and machine specifications into the problem formulation. The following three objectives were targeted for minimization: trim loss, over rolls and cutting time, and a weighted sum method was applied to these objectives.

The primary contribution of this study is the development of an effective solution approach, particularly on optimizing the cutting of ARLs into smaller bars of specific lengths as per customer orders. The approach integrates machine constraints, such as the cooling bed sizes, jagged ends, stopper and saw positions. The proposed approach, namely APOA, comprises three key components: Problem representation, Problem space reduction, and Search for the optimal solution. The APOA is adaptive in that it switches between WACO and Dijkstra’s based on specific problem conditions to optimize both small and large problems efficiently. Experimental evaluations of the approach on seven datasets provided by a case study manufacturer demonstrate the suitability of the proposed APOA. The results showed that APOA guaranteed optimality for small problems and offered near-optimal solutions for large problems with manageable time and memory usage, whereas some benchmark algorithms, such as ACO and rkGA, found optimal solutions of merely certain problems, or could not found a feasible solution within the time limit.

The benefits of the proposed approach could be demonstrated by the results from the numerical experiments. The cost saving from the seven product groups ranges from USD 1.65 – 19.24 per ton, or equivalent to 20.4% – 80.2%. This saving is significant since the steel industry is currently a highly price sensitive industry. Furthermore, the proposed solution process is versatile and can be extended to various domains beyond the steel industry, including paper roll, metal rod and wood plank cutting. In further studies, it may be beneficial to utilize substitutable PG stocks that have optional low inventory turnover because the cutting pattern production partially relies on the given PG stocks. Hence, the slow-moving inventory can be reduced while serving the order requirements.

## References

[1] Baykasoğlu A, Özbel BK. Modeling and solving a real-world cutting stock problem in the marble industry via mathematical programming and stochastic diffusion search approaches. Computers & Operations Research. 2021;128:105173. doi: 10.1016/j.cor.2020.105173

[2] Cui Y. Heuristic for the cutting and purchasing decisions of multiple metal coils. Omega. 2014;46:117–25.

[3] Fairee S, Khompatraporn C, Sirinaovakul B, Prom-On S. Trim loss optimization in paper production using reinforcement artificial bee colony. IEEE Access. 2020;8: 130647–130660.

[4] Signorini C, de Araujo S, Melega G. One-dimensional multi-period cutting stock problems in the concrete industry. International Journal of Production Research. 2022;60:2386–403.

[5] Ma N, Liu Y, Zhou Z, Chu C. Combined cutting stock and lot-sizing problem with pattern setup. Computers & Operations Research. 2018;95:44–55.

[6] Pierini L, Poldi K. An analysis of the integrated lot-sizing and cutting-stock problem formulation. Applied Mathematical Modelling. 2021;99:155–65.

[7] Oliveira W, Fiorotto D, Song X, Jones D. An extended goal programming model for the multiobjective integrated lot-sizing and cutting stock problem. European Journal of Operational Research. 2021;295:996–1007.

[8] Kim B-I, Ki Y, Son D, Bae B, Park J-S. An algorithm for a cutting problem in window frame production. International Journal of Production Research. 2016;54(14):4327–39. doi: 10.1080/00207543.2016.1148279

[9] Muter İ, Sezer Z. Algorithms for the one-dimensional two-stage cutting stock problem. European Journal of Operational Research. 2018;271(1):20–32. doi: 10.1016/j.ejor.2018.04.042

[10] Filho A, Moretti A, Pato M. A comparative study of exact methods for the bi-objective integer one-dimensional cutting stock problem. Journal of the Operational Research Society. 2018;69:91–107.

[11] Wang W, Shi Z, Shi L, Zhao Q. Integrated optimisation on flow-shop production with cutting stock. International Journal of Production Research. 2018;57(19):5996–6012. doi: 10.1080/00207543.2018.1556823

[12] Gradišar M, Resinovič G, Kljajić M. Evaluation of algorithms for one-dimensional cutting. Computers & Operations Research. 2002;29(9):1207–20. doi: 10.1016/s0305-0548(01)00025-9

[13] Ren K, Jia L, Huang J, Wu M. Research on cutting stock optimization of rebar engineering based on building information modeling and an improved particle swarm optimization algorithm. Developments in the Built Environment. 2023;13:100121.

[14] Marler RT, Arora JS. The weighted sum method for multi-objective optimization: new insights. Structural and Multidisciplinary Optimization. 2010;41(4):853–62. doi: 10.1007/s00158-010-04624-6

[15] Campello B, Ghidini C, Ayres A, Oliveira W. A multiobjective integrated model for lot sizing and cutting stock problems. Journal of the Operational Research Society. 2020;71:1466–78.

[16] Levine J, Ducatelle F. Ant colony optimization and local search for bin packing and cutting stock problems. Journal of the Operational Research Society. 2004;55(6):705–16.

[17] Gilmore P, Gomory R. A linear programming approach to the cutting stock problem—Part II. Operations Research. 1963;11(4):863–88.

[18] Armbruster M. A solution procedure for a pattern sequencing problem as part of a one-dimensional cutting stock problem in the steel industry. European Journal of Operational Research. 2002;141(2):328–40. doi: 10.1016/s0377-2217(02)00128-5

[19] Rietz J, Dempe S. Large gaps in one-dimensional cutting stock problems. Discrete Applied Mathematics. 2008;156(10):1929–35. doi: 10.1016/j.dam.2007.08.052

[20] Cerqueira GRL, Aguiar SS, Marques M. Modified Greedy Heuristic for the one-dimensional cutting stock problem. J Comb Optim. 2021;42(3):657–74. doi: 10.1007/s10878-021-00695-4

[21] Birgin EG, Romão OC, Ronconi DP. A forward-looking matheuristic approach for the multi-period two-dimensional non-guillotine cutting stock problem with usable leftovers. Expert Systems with Applications. 2023;223:119866.

[22] Araújo K, Bonates T, Prata B, Pitombeira-Neto A. Heterogeneous prestressed precast beams multiperiod production planning problem: modeling and solution methods. Transactions in Operations Research. 2021;29:660–93. doi: 10.1007/s11750-020-00589-4

[23] Pitombeira-Neto AR, Prata B de A. A matheuristic algorithm for the one-dimensional cutting stock and scheduling problem with heterogeneous orders. Transactions in Operations Research . 2019;28(1):178–92. doi: 10.1007/s11750-019-00531-3

[24] Taetragool U, Sirinaovakul B, Achalakul T. NeSS: A modified artificial bee colony approach based on nest site selection behavior. Applied Soft Computing. 2018;71:659–71.

[25] Guo Y, Song R, Zhang H, Wang C, Shin JG. A hybrid algorithm for the variable-sized bin-packing problem of pipe cutting in offshore platform construction. Journal of Marine Science and Technology. 2022; 1–17.

[26] Dorigo M, Di Caro G. Ant colony optimization: a new meta-heuristic. Proceedings of the 1999 congress on evolutionary computation-CEC99 (Cat No 99TH8406). IEEE; 1999. pp. 1470–1477.

[27] Mellouli A, Mellouli R, Masmoudi F. An innovative genetic algorithm for a multi-objective optimization of two-dimensional cutting-stock problem. Applied Artificial Intelligence. 2019;33:531–47.

[28] Ravelo SV, Meneses CN, Santos MO. Meta-heuristics for the one-dimensional cutting stock problem with usable leftover. Journal of Heuristics. 2020;26:585–618.

[29] Luo Q, Du B, Rao Y, Guo X. Metaheuristic algorithms for a special cutting stock problem with multiple stocks in the transformer manufacturing industry. Expert Systems with Applications. 2022;210:118578. doi: 10.1016/j.eswa.2022.118578

[30] Pitombeira-Neto AR, Murta AHF. A reinforcement learning approach to the stochastic cutting stock problem. EURO Journal on Computational Optimization. 2022;10:100027. doi: 10.1016/j.ejco.2022.100027

[31] Jouglet A, Carlier J. Dominance rules in combinatorial optimization problems. European Journal of Operational Research. 2011;212(3):433–44. doi: 10.1016/j.ejor.2010.11.008

[32] Dijkstra E. A note on two problems in connexion with graphs. Numerische Mathematik. 1959.;1:269–71.

[33] Fowler M. Patterns of Enterprise Application Architecture: Pattern Enterpr Applica Arch. Addison-Wesley; 2012.

[34] Gen M, Lin L. A new approach for shortest path routing problem by random key-based GA. Proceedings of the 8th Annual Conference on Genetic and Evolutionary Computation. New York, NY, USA: Association for Computing Machinery; 2006. pp. 1411–1412. doi: 10.1145/1143997.1144220

[35] Akiba T, Sano S, Yanase T, Ohta T, Koyama M. Optuna: A Next-generation Hyperparameter Optimization Framework. Proceedings of the 25th ACM SIGKDD International Conference on Knowledge Discovery and Data Mining. 2019.

